# Cognitive profiles in children and adolescents with Down syndrome

**DOI:** 10.1038/s41598-022-05825-4

**Published:** 2022-02-04

**Authors:** Sara Onnivello, Francesca Pulina, Chiara Locatelli, Chiara Marcolin, Giuseppe Ramacieri, Francesca Antonaros, Beatrice Vione, Maria Caracausi, Silvia Lanfranchi

**Affiliations:** 1 Department of Developmental Psychology and Socialization, University of Padova, Via Venezia 8, 35131 Padova, Italy; 2 IRCCS, St. Orsola-Malpighi Polyclinic, Via Massarenti 9, 40138 Bologna, Italy; 3 Department of Experimental, Diagnostic and Specialty Medicine (DIMES), Unit of Histology, Embryology and Applied Biology, University of Bologna, Via Belmeloro 8, 40126 Bologna, Italy

**Keywords:** Psychology, Human behaviour

## Abstract

The Down syndrome (DS) phenotype is usually characterized by relative strengths in non-verbal skills and deficits in verbal processing, but high interindividual variability has been registered in the syndrome. The goal of this study was to explore the cognitive profile, considering verbal and non-verbal intelligence, of children and adolescents with DS, also taking into account interindividual variability. We particularly aimed to investigate whether this variability means that we should envisage more than one cognitive profile in this population. The correlation between cognitive profile and medical conditions, parents’ education levels and developmental milestones was also explored. Seventy-two children/adolescents with DS, aged 7–16 years, were assessed with the Wechsler Preschool and Primary Scale of Intelligence-III. Age-equivalent scores were adopted, and Verbal and Non-Verbal indices were obtained for each individual. The cognitive profile of the group as a whole was characterized by similar scores in the verbal and non-verbal domain. Cluster analysis revealed three different profiles, however: one group, with the lowest scores, had the typical profile associated with DS (with higher non-verbal than verbal intelligence); one, with intermediate scores, had greater verbal than non-verbal intelligence; and one, with the highest scores, fared equally well in the verbal and non-verbal domain. Three cognitive profiles emerged, suggesting that educational support for children and adolescents with DS may need to be more specific.

## Introduction

Down syndrome (DS) is a neurogenetic disorder that affects approximately 1 in every 800 live births^1^. The most constant and typical features of DS are intellectual disability and craniofacial dysmorphisms, though many other signs and symptoms in a variety of organs and systems are being identified^2^. A specific cognitive profile has been associated with the syndrome, individuals with DS being most likely to have more pronounced language and verbal memory challenges, and relatively stronger non-verbal abilities and implicit memory skills^3,4^. More recently, a jagged profile has also emerged within each of these domains. Better receptive than expressive skills have been found in the verbal domain^5^. For example, when Deckers et al.^6^ administered the MacArthur communicative development inventories to a group of 2- to 7.9-year-old children with DS to examine their expressive and receptive vocabulary, the results showed that the children understood more words than they were able to pronounce. Receptive vocabulary can be considered a relative strength in children with DS, but the depth of their vocabulary (the extent to which word meanings have been refined and semantic knowledge has been elaborated) is not on a par with that of typically-developing (TD) peers matched on breadth of receptive vocabulary (the number of phonological entries within the lexicon that can be mapped to the correct semantic representations)^7^. As for the expressive domain, children with DS have been found weak on phonology, grammar and syntax, while their intentional use of communication and gestures, and their social use of communication generally seem to be in line with their mental age (MA)^8,9^. A pattern of strengths and weaknesses has been observed in the non-verbal domain as well. For instance, recalling locations and closure (combining different pieces of information into larger wholes, and separating larger wholes into smaller parts) have emerged as relative weaknesses in children with DS. Their ability to mentally rotate 2D and 3D objects seems to depend on the degree of rotation: individuals with DS were found capable of mental rotation but not beyond 135°^10,11^, and their performance tended to be worse than in TD children matched for MA^11^. They reveal relative strengths in spatial-sequential working memory (memory for information presented sequentially), and impairments in spatial-simultaneous working memory (memory for patterns describing spatial locations presented simultaneously)^12^. Visuo-motor integration, as assessed with a figure-copying task in which participants were asked to reproduce a series of increasingly difficult shapes, emerged as a relative strength as well^13^. As concerns visual processing, individuals with DS were found better at processing stimuli globally than locally e.g.,^14^, although this might depend on the type of task administered^15^.

In a clinical setting, cognitive functioning is typically assessed through a standardized and comprehensive intelligence test that allows the performance of a given child to be compared with that of TD children. An intelligence test is composed of several tasks (or subtests) that assess specific cognitive functioning domains and enable a general intelligence score to be computed, which is usually expressed in terms of intellectual quotient (IQ) or MA. These scores are intended to serve as a measure of an individual's intellectual abilities and potential. Studies considering general intelligence scores show a slower development in children with DS than in TD peers, with the gap between these two populations increasing with age^16^. Usually, together with a score describing general intelligence level, the majority of intelligence tests enable specific indices to be computed. The more widely used are: the “verbal index”, which assesses the ability to access and apply acquired word knowledge and the capacity for reasoning that involves words; and the “non-verbal (or performance) index”, which assesses the ability to understand and apply visuo-spatial information, and the capacity for visuo-spatial reasoning. In recent years, other indices have been proposed that describe other aspects of intelligence, such as working memory or processing speed e.g.,^17^. In the present study we focus on verbal and non-verbal indices because they are the most widely used in intelligence tests.

Although not many studies directly explored the profile of verbal and non-verbal intelligence in individuals with DS, more information is available in the literature because many studies reported verbal and non-verbal intelligence indices for descriptive purposes (though they were not the focus of the study). The data available paint a varied picture. Considering the general profile reported in individuals with DS, which describes better non-verbal than verbal skills, we could expect this to apply to verbal and non-verbal intelligence indices too. Some studies accordingly documented an intelligence profile characterized by greater non-verbal than verbal abilities e.g.,^7–9^. For example, Lanfranchi and colleagues^12^ used the Wechsler Preschool and Primary Scale of Intelligence (WPPSI) with a group of children with DS aged 8–19, and found higher scores on the non-verbal index (MA M = 61, SD = 10.4) than on the verbal one (MA M = 51.1, SD = 6.5). Similar results were reported by Perez Duarte and colleagues^18^, using the versions of the Wechsler scales for older individuals (the Wechsler Intelligence Scale for Children, WISC, and Wechsler Adult Intelligence Scale, WAIS). Their sample with DS aged 7–18 had a verbal IQ of 52.6 (SD = 7.3) and a non-verbal IQ of 55.2 (SD = 9.7). Finally, in a study by Breslin et al.^19^, a group of 7- to 12-year-old individuals with DS and associated Obstructive Sleep Apnea (OSA) scored higher on the non-verbal intelligence index (IQ M = 48.53, SD = 9.92) than on the verbal index (IQ M = 45.11, SD = 8.83), when assessed with the Kaufman Brief Intelligence Test (KBIT-2). Neither of these studies focused on exploring the cognitive profile associated with DS, however, so no direct comparisons were drawn to ascertain the significance of the discrepancies between the indices. Other studies found higher scores for verbal than for non-verbal indices, however^19–22^. For example, still in the Breslin et al. study^19^, a second group of individuals with DS (without OSA) scored slightly higher on a verbal index (IQ M = 54.42, SD = 11.54) than on a non-verbal index (IQ M = 52.67, SD = 13.55). In another study—one of the few deliberately comparing intelligence indices—Pezzuti et al.^20^ analyzed the intellectual profile of children and adolescents with DS (aged 7–16 years) using the WISC-IV. The highest scores were recorded in the Verbal Comprehension Index (IQ M = 42.70, SD = 17.71), with intermediate scores in the Perceptual Reasoning (IQ M = 38.87, SD = 16.57) and Processing Speed (M IQ = 37.53, SD = 11.91) indices, and the lowest scores for Working Memory (IQ M = 31.31, SD = 17.21). Specifically focusing on the indices of interest here, Pezzuti et al.^20^ found scores significantly higher on a verbal index (Verbal Comprehension Index) than on a non-verbal index (Perceptual Reasoning index), with a medium effect size. Strengths and weaknesses emerged within a given index as well. In the Verbal Comprehension subtests, performance was stronger in the Similarities task, and weaker in the Vocabulary and Comprehension tasks. In the subtests measuring Processing Speed, a relative strength emerged in the Block Design task. The participants’ performance was comparable in the subtests generating the other two indices. Similarly, Evans and Uljarevic^21^ found that children and adolescents with DS tended, at both 7 and 15 years of age, to have a higher verbal than non-verbal IQ when assessed with the Stanford-Binet Intelligence Scale-4th edition. In particular, the group of children had a verbal IQ of 58.21 (SD = 10.61) and a non-verbal IQ of 53.23 (SD = 8.06), while the group of adolescents had a verbal IQ of 49.09 (SD = 6.58) and a non-verbal IQ of 47.27, (SD = 9.57). Higher scores in the verbal domain also emerged in a study by Sabat et al.^22^, who examined a sample of adolescents with DS (aged 12–18 years) using the WISC or WAIS. Here again, their verbal IQ (M = 48.36, SD = 5.94) was higher than their non-verbal IQ (M = 46.28, SD = 3.79), though the researchers did not define the magnitude of this difference because this was not the aim of the study. Finally, a third set of studies described homogeneous verbal and non-verbal intelligence indices in individuals with DS. For instance, Cebula et al.^23^ used the WPPSI to assess a group of children/adolescents with DS (aged 9–18 years), and found that their verbal and non-verbal MA scores were much the same (M = 4.07, SD = 7.2 and M = 4.48, SD = 7.0 respectively).

In short, considering the two intelligence indices, some studies find the more generally-acknowledged profile associated with DS, i.e., a better non-verbal than verbal intelligence, while some find the opposite, with a better verbal than non-verbal intelligence, and some delineate a balanced cognitive profile. This heterogeneous picture highlights the great variability seen in individuals with this syndrome. As Karmiloff-Smith and colleagues^24^ underscored, the wide range of individual differences at every descriptive level (genetic, cellular, neural, cognitive, behavioral, and environmental) could help to explain the diverse cognitive profiles encountered in DS e.g.,^24–27^. When they considered cognitive and behavioral scores, Karmiloff-Smith and colleagues^24^ found the standard deviation in groups with DS higher than in typically-developing samples e.g.,^28^, and a wide range of scores e.g.,^29,30^. The issue of cognitive interindividual variability focusing on verbal and non-verbal indices in DS was addressed by Tsao and Kindelberg^26^. They assessed 88 children aged from 5.11 to 11.8 years, using the Differential Scales of Intellectual Efficiency (EDEI-R), which is a measure of verbal and non-verbal reasoning abilities. Verbal performance was assessed in terms of vocabulary (picture naming), knowledge (acquired in the course of everyday life or at school), and social comprehension (social adaptive behaviors, with questions focusing on comprehension of interpersonal relations). Non-verbal performance was assessed on classification (finding a principle of similarity between two familiar objects), categorical analysis (arranging geometric forms by shape, color and dimension), and practical adaptation (embedding test). The results of their study confirmed a marked interindividual variability, and a clustering approach led to four cognitive profiles being identified, each featuring a particular pattern of abilities. Four different sub-groups emerged: one (n = 22) characterized by similar scores on verbal and non-verbal indices, and by a score for classification that was very low compared with the scores obtained in the other two non-verbal tests; a second (n = 24) performed poorly in both areas, but especially on the verbal index; a third (n = 22) scored significantly higher on the verbal than on the non-verbal index, with the lowest scores in the tasks involving categorical analysis and practical adaptation; and a fourth (n = 20) scored higher on the non-verbal index, and highest for practical adaptation. This variability might be attributable to individual and environmental factors having an impact on a child’s development. Some such factors could be medical conditions, developmental trajectories and parents’ education levels. Medical conditions have been shown to influence cognition in DS: congenital heart defects seem to account for a portion of the variance in the severity of language impairments^31,32^, and a history of gastrointestinal surgery has been associated with this diagnostic group’s cognitive^23^ and motor development^33^. Moreover OSA associated with DS might also reflect on both the severity of impairments and cognitive profiles^19^. Another issue concerns when individuals with DS reach certain developmental milestones, and their developmental trajectories can be charted vis-à-vis their acquisition of earlier stages of development. It has been demonstrated that the delay with which individuals with DS reach these milestones is linked to their subsequent cognitive and language development e.g.,^34–37^. Motor milestones have been found related to subsequent cognitive and language development^37^, and communication milestones to language development^36^. Finally, parents’ education is also an important factor contributing to children’s development. Just as research on typical development identified a strong association between mothers’ education levels and their children’s cognitive development^38^, this also applies to DS: those whose mothers had a better education scored higher in the Stanford-Binet tasks^39^.

On the topic of assessing intelligence in individuals with DS, there are some methodological aspects to consider. It has been demonstrated that using a test appropriate for chronological age frequently leads to a floor effect that is not informative^20^. Following a procedure already used in the field e.g.,^40,41^, we opted in the present study to assess intelligence using a test consistent with the MA of the sample, the WPPSI-III. This enabled us to completely avoid the floor effect, though it prevented us from computing standardized scores to compare the performance of our DS sample with TD peers. See Pulina et al.^42^ for a more comprehensive discussion. It was nonetheless possible to compute age-equivalent (AE) scores that, though less robust from a statistical standpoint, have proved sufficiently reliable and informative in the field of intellectual disability e.g.,^43^.

Bearing in mind the previous literature, and considering the paucity of studies that focused directly on comparing verbal and non-verbal intelligence, our study aims to explore and compare verbal and non-verbal intelligence indices, in a sample of children and adolescents with DS, also considering interindividual variability. In addition, in the light of studies showing the variability within the two domains, our goal is also to explore the DS profile in terms of performance within the verbal and non-verbal indices. In particular, we aim to:

1. explore and compare verbal and non-verbal intelligence, as assessed with the WPPSI-III, in a sample of individuals with DS. Although many studies report verbal and non-verbal intelligence indices for descriptive purposes, few directly focus on comparing these two aspects to shed light on the intelligence profile associated with DS. Based on the generally-described greater strength in non-verbal than in verbal skills, we might expect individuals with DS to have higher scores in the non-verbal domain than in the verbal one. That said, we could envisage other profiles, and a broader and more varied picture, in the light of previous research conducted using cognitive assessment batteries. It is also worth exploring the profile within the two domains to see if peaks and troughs emerge from the subtests for each domain. Within the verbal domain, we expect higher scores on receptive than on expressive vocabulary e.g.,^9^. A jagged profile might emerge within visuo-spatial subtests too;
2. examine whether, considering verbal and non-verbal intelligence indices, the variability that emerged from different studies could also be found in our sample. In other words, we want to explore whether interindividual variability in our sample could be classified in terms of subgroups of participants with different cognitive profiles. Considering the results reached by Tsao and Kindelberg^26^, we could expect to identify some individuals with the better non-verbal than verbal skills, but also subgroups of children with other profiles. Tsao and Kindelberg assessed intelligence using the EDEI-R scale, however. Finding similar results using a different intelligence test would support the presence of different cognitive sub-profiles, whereas different results would suggest that the profiles emerging in the Tsao and Kindelberg study are more linked to the intelligence test they used. If more cognitive sub-profiles emerge, we will explore the children’s performance within the verbal and non-verbal indices in each group;
3. explore the possibility of an association between a particular cognitive profile and other variables, such as medical conditions (we separately consider congenital heart defects, a history of heart surgery, and OSA), and parents’ education level. The results reported by Breslin^19^, for example, would suggest a profile characterized by a higher non-verbal than verbal index in children with DS and associated OSA;
4. investigate differences between subgroups in the age of reaching milestones such as babbling, sitting, first words and walking, as reported by parents; moreover correlate the age of reaching milestones and the WPPSI global score in each group.

We consider important to explore all these aspects because a better understanding of the different cognitive facets of DS could lead to more effective intervention programs, more specifically tailored to a given individual’s strengths and vulnerabilities.

## Method

### Participants

The study concerned 72 children and adolescents with DS (males, n = 41). Their mean chronological age (CA) in months was 134.38 (SD = 31.24, min = 85.00 and max = 195.00). Participants came from all over Italy and were recruited during their annual visit to the Neonatology Unit at St. Orsola-Malpighi Polyclinic in Bologna (Italy). The inclusion criteria were: a diagnosis of DS with homogeneous or mosaic trisomy 21; and a CA ranging between 7 and 17 years. This age range was chosen because it coincides with the time when individuals with DS reach their maximum developmental level, usually with no decline due to genetic or environmental factors. Trisomy 21 was confirmed by a karyotype analysis with amniocentesis during a prenatal screening, or by a blood sample after birth. None of the participants had mosaic trisomy. All participants were attending mainstream schools.

### Measures

#### Cognitive assessment

All participants were assessed with the Wechsler Preschool and Primary Scale of Intelligence-III, WPPSI-III^17^, a standardized method for measuring cognitive development for preschoolers and young children (aged from 2.6 to 7.3 years). Although the WPPSI-III was designed for young children, it was used here to avoid any floor effect and because it was considered more in line with the supposed mental age of our sample. This approach has already been adopted in previous studies e.g.,^26,40,41^. The WPPSI has two versions, one for younger children (from 2.6 to 3.11 years old), and the other for older children (from 4 to 7.3 years of age). The former was used in the present study to ensure that all the children and adolescents could understand and complete the tasks. The latter would have led to a floor effect for several participants, which would have told us nothing about their skills. We also judged it more appropriate to derive performance indices from a test designed for younger children, rather than to obtain them statistically from a floor performance. Although we chose to administer subtests intended for a younger age range, these tests could be used with children in the older age range to derive AE scores from the normative data. The version for younger children includes 5 subtests: Receptive Vocabulary, Picture Naming, Information, Block Design, and Object Assembly. In Receptive Vocabulary (which assesses receptive language), respondents are asked to look at a group of four pictures, and to point to the one the examiner names aloud. In Picture Naming (designed to measure expressive vocabulary), they have to name pictures shown one at a time in a stimulus booklet. In the Information subtest (for assessing a child’s ability to acquire, retain, and retrieve general factual knowledge), respondents are asked questions testing their general knowledge. The Block Design subtest (which measures the ability to analyze and synthesize abstract visual stimuli, and to form non-verbal concepts) involves participants having to reproduce models with a set of blocks. Finally, there is Object Assembly (which assesses visual-perceptual regulation, and the ability to analyze and synthesize an abstract design), where participants are shown pieces of a puzzle in a standard arrangement and asked to fit the pieces together to form a given figure. The minimum score that a child could obtain in each subtest was 0, while the maximum was 38 for Receptive Vocabulary, 30 for Picture Naming, 34 for Information, 40 for Block Design, and 37 for Object Assembly. The scores obtained in the subtests were used to calculate a Verbal Index (which includes Receptive Vocabulary, Picture Naming and Information), and a Non-Verbal Index (comprising Block Design and Object Assembly). A Total Index can be calculated as well. Using a test standardized for younger children prevented us from considering standard scores, so AE scores were used instead, following a procedure suggested by Toffalini et al.^43^. In their paper, Toffalini et al.^43^ compared AE and Z scores—calculated following the recommendations of Orsini et al.^44^—and they demonstrated that the two methods were equally effective in preventing floor effects. The authors suggested that AE scores might be more readily interpreted than Z, however, and therefore of greater clinical value. The norms reported in the WPPSI-III manual were used to convert raw scores into AE scores. The normative data do not cover all the range of raw scores, however, so—for each subtest—we calculated a regression slope that enabled us to estimate the missing values with the following formula: AE score = slope × raw score + intercept. Since AE scores are influenced by CA, they were partialized for CA, and their residuals were used in the analyses. We opted to report AE scores in the descriptive statistics, however, as they are more readily interpretable.

#### Participants’ developmental h﻿istory

Caregivers provided family background and information on their children’s development, including any medical conditions, when they reached the main milestones, and whether they attended any intervention programs. For the purposes of the present study, we considered the age when they reached specific milestones (sitting, babbling, walking, and first words), medical conditions (heart problems, a history of heart surgery and OSA), and parents’ education level.

### Procedure

The data considered here were collected as part of a broader project exploring the correlation between genotype and phenotype in DS. All participants were attending the Day Hospital at the Neonatology Unit, Sant’Orsola-Malpighi Polyclinic, Bologna, and the study was proposed at a routine annual follow-up for cases of DS. Written informed consent was obtained from all participants’ parents/caregivers. Then the children and adolescents were assessed at the Department of Developmental Psychology at the University of Padova. The assessment sessions lasted approximately 90 min. Participants were recruited between November 2017 and February 2020. The present study was approved by the independent Ethics Committee at the St. Orsola-Malpighi Polyclinic and University Hospital (Bologna, Italy) and it was performed in accordance with the Declaration of Helsinki.

### Analysis plan

To explore participants’ overall cognitive profile, descriptive statistical analyses were conducted on the WPPSI verbal and non-verbal indices and subtests, then two ANOVAs were run on the scores obtained, one for the indices, and the other for the subtests.

To identify any subgroups with different cognitive profiles, a cluster analysis was run using the WPPSI Verbal and Non-Verbal Indices. Cluster analysis is an exploratory statistical method used to identify naturally-occurring groups or patterns of responses in a given set of measures or scales. Participants were empirically sorted into groups based on their relative similarities to one another on the measures considered^45^. AE scores for the two indices were partialized for CA, and their residuals were used in the analyses. The residuals then underwent hierarchical cluster analysis, using squared Euclidean distances to distinguish the clusters. The agglomeration method was used because the Agglomerative Coefficient (AC) indicated that Ward’s method was the one capable of identifying the strongest clustering structures (“Average” AC 0.89; “Single” AC 0.68; “Complete” AC 0.95; “Ward” AC 0.97; where values closer to 1 suggest a more balanced clustering structure, and those closer to 0 suggest less well-formed clusters). The “NbClust” package in R was used to validate the results of clustering analysis. Since this package provides an exhaustive list of validity indices for estimating the number of clusters in a data set^46^, it was possible to compare the clusters resulting from the hierarchical cluster analysis with 30 fit indices. A majority rule approach was considered to facilitate the choice of clusters in the real data sets^46^. To further confirm the results, the “tidyLPA” package in R was used to check the fit statistics on models with one to four clusters. The Bayesian Information Criterion (BIC)^47^, the Entropy value, and the Bootstrapped Likelihood Ratio Test (BLRT) were considered. When the BIC is applied, lower values indicate a better fit. The Entropy value gives an indication of a model’s classification quality, with values ranging from 0 to 1; higher values indicate a better classification quality^48^, and values above 0.80 are generally assumed to indicate an adequate classification quality^49^. The BLRT compares the improvements in fit between neighboring class models (i.e., a model with k clusters to a model with k−1 clusters), generating a p value that is useful for establishing whether including one more class leads to a statistically significant improvement in the fit.

Two repeated-measures ANOVAs were used to explore the profiles between and within clusters, one considering the indices, the other considering the subtests, with Cluster as the between-subjects factor and Index/Subtest as within-subject factors.

When the assumption of sphericity was violated in the ANOVAs, the Greenhouse–Geisser adjustment was applied to p values (reported as p_[gg]_). Post-hoc t-tests were two-tailed and the p values were corrected for multiple comparisons using the Bonferroni method (i.e., the value of alpha divided by the number of comparisons). Cohen’s d was calculated to ascertain the magnitude of the difference between the clusters at each session. We also report Bayes factors (BF_10_) expressing the probability of the data, given H1 relative to H0 (i.e., values larger than 1 are in favor of H1, and those smaller than 1 are in favor of H0). The cut-offs for the BFs are: “anecdotal” (BF < 3), “moderate” (BF > 3), “strong” (BF > 10), “very strong” (BF > 30), or “extreme” (BF > 100) (Jeffreys, 1961). ANOVAs were run with AE scores partialized for CA, and their residuals were used. These analyses were also run on AE scores with CA as the control variable to see for any differences emerged. The results led to the same conclusion, so those with the residuals are reported for consistency with the cluster analysis where these scores were used.

Finally, to test the association with other variables, the chi-squared test was run for categorical variables, and correlations for continuous variables. Since the three medical conditions considered were dichotomous variables (present vs absent), and so was parents’ education level (≤ high school vs > higher education), the chi-squared test was conducted in these cases, while correlations were run for age on reaching milestones.

Analyses were run using R (Version 4.0.0)^50^.

## Results

### Defining the cognitive profile of the sample as a whole

Descriptive statistics for each subtest and for the Verbal, Non-Verbal and Total indices are given in Table 1. No children performed at the floor for any subtest, while only one child performed at the ceiling only for the Object Assembly subtest.

**Table 1:** Descriptive statistics for the sample as a whole.

	Raw scores M (SD) [min–max]	Age-equivalent scores M (SD) [min–max]
**Verbal index**		49.95 (18.33) [3.27–85.86]
Receptive Vocabulary	21.11 (7.20) [1–34]	50.80 (20.52) [3.00–88.08]
Picture Naming	16.96 (6.20) [3–29]	49.87 (20.43) [3.82–89.58]
Information	19.38 (6.80) [3–33]	49.18 (21.21) [3.00–92.32]
**Non-verbal index**		47.21 (14.11) [17.47–81.29]
Block Design	18.90 (6.04) [3–34]	47.16 (18.44) [3.00–93.77]
Object Assembly	14.14 (8.00) [2–37]	47.25 (13.41) [26.91–85.57]
**Total index**		48.85 (18.26) [12.82-80.09]

**Figure 1:**
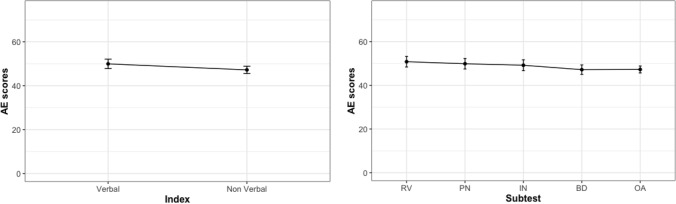
Cognitive profile of the sample as a whole, considering indices and subtests. *RV*=Receptive Vocabulary, *PN* = Picture Naming, *IN =*  Information, *BD =* Block Design, *OA =* Object Assembly.

The profile of the sample as a whole was investigated by running two ANOVAs, one with Index as the within-subject factor, the other with Subtest as the within-subject factor. No effect of Index (p = 1.000, η_p_^2^ < 0.001, BF_10_ = 0.17) or Subtest emerged (p_[gg]_ = 1.000, η_p_^2^ < 0.001, BF_10_ = 0.006), suggesting a flat cognitive profile. The standard deviations were high, however, suggesting a marked interindividual variability. The profile of the sample as a whole is graphically represented in Fig. 1, where Indices and Subtests are considered separately.

### Identifying clusters

Hierarchical cluster analysis using Ward’s method and Euclidean distances resulted in the following indices: 9 indices pointed to two as the best number of clusters, while 10 indicated three, and 4 suggested four. Taking the majority rule approach, the best number of clusters was three (C1, C2, C3).

To confirm as much, models with one to four clusters were run on the latent profiles analysis, which revealed that the 3-cluster solution provided the best overall model fit for the data, confirming the assumption of a heterogeneous developmental picture within the sample of children/adolescents with DS that could be identified with the aid of mixture modelling. The results are presented in Table 2.

**Table 2:** Comparison of overall model fit statistics for latent profiles analysis considering 1–4 clusters.

	Overall model fit			
	1 cluster	2 clusters	3 clusters	4 clusters
BIC	1204.59	1204.15	1204.34	1214.58
Entropy		0.55	0.85	0.87
BLRT (p value)		0.02	0.03	0.28

*BIC* Bayesian information criterion, *BLRT*  bootstrapped likelihood ratio test.

Though the 3-cluster solution did not have the lowest BIC, it did have a better entropy value than the 2-cluster solution. On examining the BLRT findings it emerged that the 3-cluster model showed a better fit than the 2-cluster model, and there was no additional improvement in the fit with the 4-cluster model.

Although residuals were adopted in the analyses, descriptive statistics are reported for AE scores as they are more readily interpretable (Table 3). The three groups of participants were labeled as follows: C1, the Verbal Profile group (scoring higher on verbal than non-verbal index); C2, the Non-Verbal Profile group (scoring higher on non-verbal index); and C3, the Homogeneous Profile group (with similar verbal and non-verbal indices). The three groups were similar in terms of the numbers of participants in each one. No significant differences emerged between the three groups in terms of CA (p > .05, BF10 = 0.12).

**Table 3:** Descriptive statistics for the three clusters (AE scores).

	Verbal profile (n = 29) M (SD)	Non-verbal profile (n = 22) M (SD)	Homogeneous profile (n = 21) M (SD)
**Verbal index**	55.80 (10.59)	29.36 (11.02)	63.44 (14.20)
Receptive vocabulary	55.64 (17.11)	33.00 (14.57)	62.78 (18.14)
Picture naming	56.37 (13.45)	27.66 (14.36)	64.14 (13.92)
Information	55.38 (14.10)	27.42 (16.07)	63.41 (16.08)
**Non-verbal index**	41.11 (9.38)	40.11 (11.06)	63.06 (9.06)
Block design	42.77 (15.59)	36.86 (15.48)	64.01 (13.15)
Object assembly	39.45 (8.87)	43.36 (9.56)	62.11 (9.76)
**Total index**	49.92 (8.47)	33.66 (8.34)	63.28 (11.03)
Chronological age	133.65 (31.17)	134.18 (34.52)	135.57 (29.16)

### Comparison between clusters—verbal and non-verbal indices

**Figure 2:**
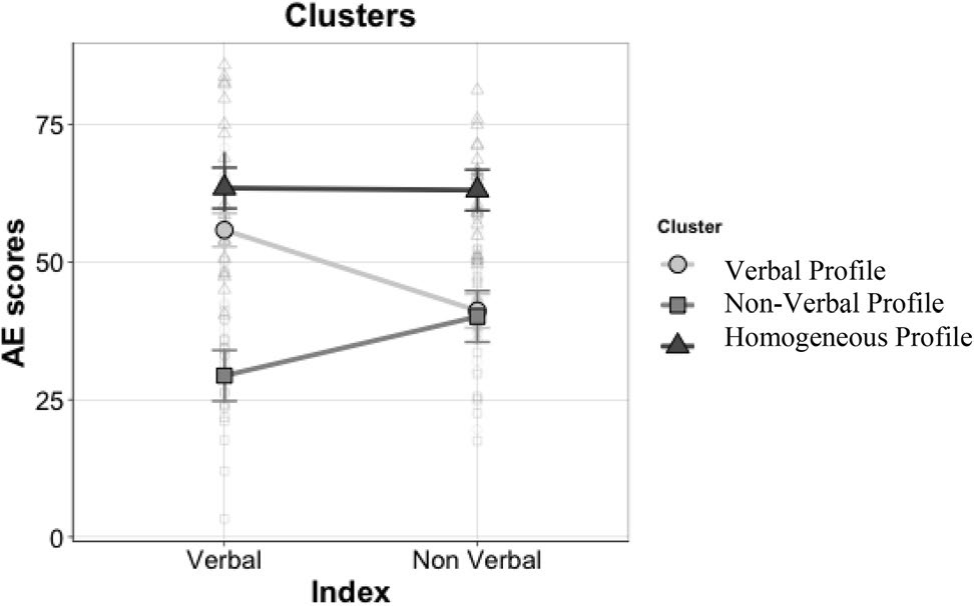
The cognitive profiles based on the indices.

The results of the repeated-measures ANOVA with Index as the within-subject variable are given in Table 4, and graphically represented in Fig. 2.

**Table 4:** Post-hoc analyses, cluster × index.

	Between-subjects comparison			Within-subject comparison		
	Verbal profile vs non-verbal profile	Verbal profile vs homogeneous profile	Non-verbal profile vs homogeneous profile	Verbal profile	Non-verbal profile	Homogeneous profile
Verbal index	t = 10.96 p < .001 d = 1.19 BF_10_ = 9.53 × 10^13^	t = −2.29 p = 0.03 d = 0.32 BF_10_ = 3.17	t = −9.88 p < .001 d = 1.40 BF_10_ = 3.03 × 10^19^	t = 5.83 p < .001 d = 0.61 BF_10_ = 6.69 × 10^3^	t = −4.36 p < .001 d = 0.60 BF_10_ = 1.12 × 10^2^	t = −0.94 p = 0.36 d = 0.10 BF_10_ = 0.34
Non-verbal index	t = 0.41 p = 0.70 d = 0.05 BF_10_ = 0.30	t = −8.13 p = < .001 d = 0.96 BF_10_ = 2.89 × 10^10^	t = −10.44 p < .001 d = 0.94 BF_10_ = 2.89 × 10^7^			

*t*  t-test value; *p* significance level; *d*  Cohen’s d expressing the effect size; *BF*_*10*_  Bayes factor expressing the probability of the data given H1 relative to H0.

A significant effect of Cluster emerged (F(2,69) = 89.51, p < 0.001, η_p_^2^ = 0.72, BF_10_ = 1.03 × 10^14^), and subsequent post-hoc analyses showed that the Homogeneous Profile group’s scores were higher than the Verbal Profile group (t = 7.07, p < 0.001, d = 0.85, BF_10_ = 3.77 × 10^7^) or the Non-Verbal Profile group (t = 11.81, p < 0.001, d = 1.57, BF_10_ = 1.72 × 10^16^). The Non-Verbal Profile group had lower scores than the Verbal Profile group (t = −6.20, p < 0.001, d = 0.83, BF_10_ = 1.52 × 10^7^). No main effect of Index was found. The Cluster × Index interaction was significant (F(2,69) = 26.93, p < 0.001, η_p_^2^ = 0.44 BF_10_ = 1.14 × 10^23^), so post-hoc analyses were run (Table 4). Using Bonferroni’s correction, we adjusted the alpha levels to 0.016 (i.e., 0.05/3) for the comparisons between groups, and to 0.025 (i.e., 0.05/2) for the comparisons between Verbal and Non-Verbal Indices within the three groups.

Considering the within-subject comparisons, the Verbal Profile group had significantly higher scores in the Verbal Index (M = 55.80) than in the Non-Verbal Index (M = 41.11); vice versa, the Non-Verbal Profile group scored significantly higher in the Non-Verbal Index (M = 40.11) than in the Verbal Index (M = 29.36); and for the Homogeneous Profile group there was no significant difference between the Verbal and Non-Verbal Indices (M = 63.44 and M = 63.06, respectively).

The between-subjects comparisons showed that: the Verbal Profile group scored significantly higher than the Non-Verbal Profile group in the Verbal Index (M = 55.80 and M = 29.36, respectively); the Non-Verbal Profile group scored significantly lower than the Homogeneous Profile group in both the Verbal Index (M = 63.44 and M = 29.36, respectively) and the Non-Verbal Index (M = 63.06 and M = 40.11, respectively); and the Homogeneous Profile group scored higher scores than the Verbal Profile group in the Non-Verbal Index (M = 63.06 and M = 41.11, respectively).

### Comparison between clusters—WPPSI subtests

**Figure 3:**
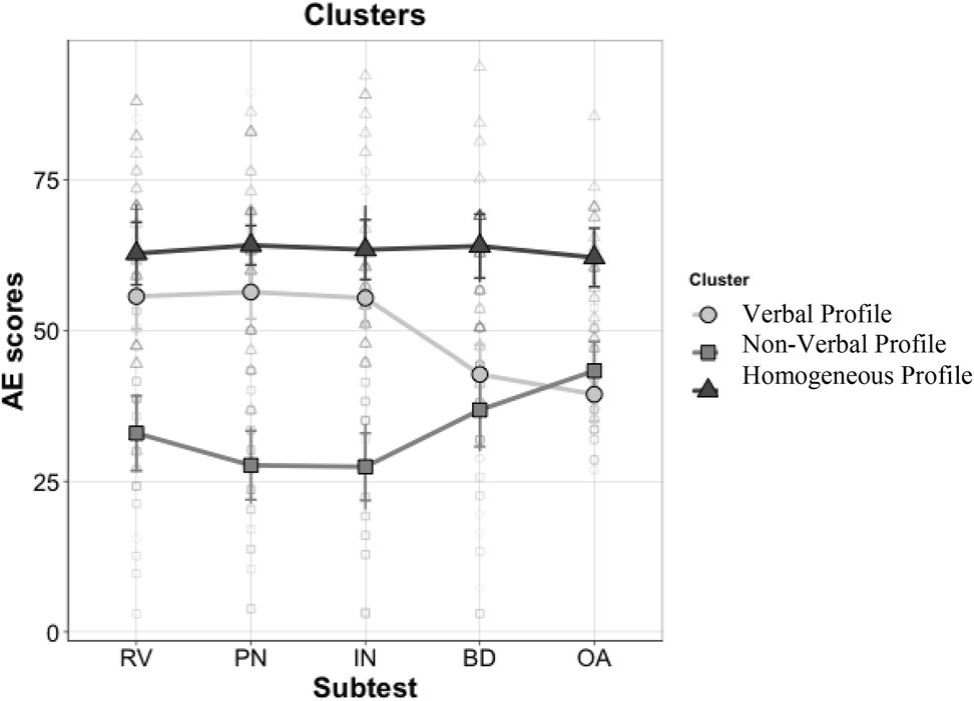
The cognitive profiles based on the subtests. *RV =* Receptive Vocabulary, *PN =* Picture Naming, *IN =*  Information, *BD =* Block Design, *OA =* Object Assembly.

The second repeated-measures ANOVA was run to examine the profiles by single subtest. The results are given in Tables 5 and 6, and graphically represented in Fig. 3.

**Table 5:** Post-hoc analyses, subtest × index—between-subjects comparison—groups paired comparisons in each subtest.

	Between-subjects comparison		
	Verbal profile vs non-verbal profile	Verbal profile vs homogeneous profile	Non-verbal profile vs homogeneous profile
**Verbal index**			
Receptive Vocabulary	t = 5.99 p < .001 d = 0.73 BF_10_ = 3.70 × 10^4^	t = −1.48 p = 0.15 d = 0.21 BF_10_ = 0.73	t = −6.45 p < .001 d = 0.88 BF_10_ = 1.16 × 10^5^
Picture Naming	t = 8.11 p < .001 d = 0.93 BF_10_ = 9.35 × 10^7^	t = −2.10 p = 0.04 d = 0.24 BF_10_ = 1.80	t = −9.04 p < .001 d = 1.07 BF_10_ = 2.22 × 10^8^
Information	t = 7.01 p < .001 d = 0.91 BF_10_ = 1.82 × 10^6^	t = −1.84 p = 0.07 d = 0.25 BF_10_ = 1.20	t = −7.83 p < .001 d = 1.07 BF_10_ = 6.53 × 10^6^
**Non-verbal index**			
Block Design	t = 1.46 p = 0.15 d = 0.18 BF_10_ = 0.69	t = −5.95 p < .001 d = 0.68 BF_10_ = 2.29 × 10^4^	t = −6.54 p < .001 d = 0.81 BF_10_ = 1.18 × 10^5^
Object Assembly	t = −1.50 p = 0.14 d = 0.13 BF_10_ = 0.71	t = −9.25 p < .001 d = 0.72 BF_10_ = 9.60 × 10^8^	t = −7.10 p < .001 d = 0.56 BF_10_ = 6.98 × 105

*t* t-test value; *p* significance level; *d*  Cohen’s d expressing the effect size; *BF*_*10*_  Bayes factor expressing the probability of the data given H1 relative to H0.

**Table 6:** Post-hoc analyses, subtest × index—within-subject comparisons. Paired comparisons between subtests within each group.

		Within-subject comparison		
		Verbal profile	Non-verbal profile	Homogeneous profile
Receptive Vocabulary	Picture Naming	t = −0.50 p = 0.62 d = 0.06 BF_10_ = 0.22	t = 1.11 p = 0.28 d = 0.14 BF_10_ = 0.39	t = −0.77 p = 0.45 d = 0.08 BF_10_ = 0.30
	Information	t = −0.36 p = 0.72 d = 0.05 BF_10_ = 0.21	t = 0.99 p = 0.33 d = 0.13 BF_10_ = 0.36	t = −0.78 p = 0.44 d = 0.08 BF_10_ = 0.30
	Block Design	t = 2.48 p = 0.02 d = 0.36 BF_10_ = 2.60	t = −1.76 p = 0.09 d = 0.25 BF_10_ = 0.83	t = −1.36 p = 0.19 d = 0.16 BF_10_ = 0.51
	Object Assembly	t = 4.32 p < .001 d = 0.48 BF_10_ = 1.56 × 10^2^	t = −3.62 p = 0.001 d = 0.45 BF_10_ = 23.74	t = −0.81 p = 0.43 d = 0.10 BF_10_ = 0.30
Picture Naming	Information	t = 0.10 p = 0.92 d = 0.01 BF_10_ = 0.20	t = −0.13 p = 0.90 d = 0.01 BF_10_ = 0.22	t = 0.005 p = 1.00 d = 0.001 BF_10_ = 0.23
	Block Design	t = 3.59 p = 0.001 d = 0.42 BF_10_ = 27.41	t = −2.55 p = 0.02 d = 0.39 BF_10_ = 2.97	t = −1.03 p = 0.31 d = 0.08 BF_10_ = 0.36
	Object Assembly	t = 4.52 p < .001 d = 0.54 BF_10_ = 2.55 × 10^2^	t = −5.39 p < .001 d = 0.60 BF_10_ = 9.96 × 10^2^	t = −0.23 p = 0.82 d = 0.02 BF_10_ = 0.24
Information	Block Design	t = 3.26 p = 0.003 d = 0.41 BF_10_ = 13.19	t = −2.82 p = 0.01 d = 0.39 BF_10_ = 4.89	t = −0.61 p = 0.55 d = 0.06 BF_10_ = 0.27
	Object Assembly	t = 4.79 p < .001 d = 0.53 BF_10_ = 4.97 × 10^2^	t = −4.53 p < .001 d = 1.46 BF_10_ = 1.61 × 10^2^	t = −0.21 p = 0.83 d = 0.02 BF_10_ = 0.23
Block design	Object Assembly	t = 1.17 p = 0.25 d = 0.11 BF_10_ = 0.36	t = −2.24 p = 0.04 d = 0.21 BF_10_ = 1.73	t = 0.59 p = 0.56 d = 0.06 BF_10_ = 0.27

*t* t-test value; *p* significance level; *d* Cohen’s d expressing the effect size; *BF*_*10*_ Bayes factor expressing the probability of the data given H1 relative to H0.

There was an effect of Cluster (F(2,69) = 90.03, p < 0.001, η_p_^2^ = 0.72, BF_10_ = 1.68 × 10^17^), and subsequent post-hoc analyses showed that the Homogeneous Profile group scored higher than the Verbal Profile group (t = 7.64, p < 0.001, d = 0.75, BF_10_ = 1.95 × 10^14^) or the Non-Verbal Profile group (t = 15.44, p < 0.001, d = 1.58, BF_10_ = 8.87 × 10^32^). The Verbal Profile group scored higher than the Non-Verbal Profile group (t = 9.02, p < 0.001, d = 0.95, BF_10_ = 9.74 × 10^9^). There was no main effect of Subtest. A Cluster x Subtest interaction emerged (F(8,276) = 9.58, p < 0.001, η_p_^2^ = 0.21, BF_10_ = 1.53 × 10^9^), so post-hoc analyses were run (see Table 5 for between-subjects comparisons, and Table 6 for within-subject comparisons). Using Bonferroni’s correction, we adjusted the alpha levels to 0.016 (i.e., 0.05/3) for comparisons between groups, and to 0.005 (i.e., 0.05/10) for comparisons between subtests within the three groups.

Between-subjects comparisons showed that the Verbal Profile group scored higher than the Non-Verbal Profile group in all the subtests contributing to the Verbal Index. No differences emerged for the Non-Verbal Index subtests. The Verbal Profile group had lower scores than the Homogeneous Profile group in all the Non-Verbal Index subtests, while the Non-Verbal Profile group scored lower than the Homogeneous Profile group in the subtests contributing to both indices.

In the within-subject comparisons, the Verbal Profile group scored lower on Object Assembly than in any of the other Verbal Index subtests, and lower on Block Design than on Picture Naming or Information. The Non-Verbal Profile group scored higher on Object Assembly than on any of the other Verbal Index subtests. The Homogeneous Profile group showed no significant differences between the subtests.

### The role of medical problems

Considering medical problems, the percentages of individuals with heart problems, a history of heart surgery and OSA are given in Table 7.

**Table 7:** Prevalence of medical conditions in each group.

	Verbal (N = 29)	Non-verbal (N = 22)	Homogeneous (N = 21)	N condition/N whole sample^a^
Heart problems % yes (n)	37% (15)	30% (12)	32% (13)	40/64
Prior heart surgery % yes (n)	30% (6)	40% (8)	30% (6)	20/63
OSA % yes (n)	29% (5)	29% (5)	42% (7)	17/56

^a^Data on the presence of these conditions were not available for some participants.

The chi-squared test revealed no associations between any of these medical conditions and group (p < 0.05).

### The role of mothers’ education

Table 8 shows the percentage of mothers' with the different education levels in each subgroup. A chi-squared test exploring the association between parents’ education levels and group revealed no association between the mothers’ education level and the groups.

**Table 8:** Prevalence of parents with an education level higher than high school.

	Verbal (N = 29)	Non-verbal (N = 22)	Homogeneous (N = 21)	N condition/N whole sample
Mothers’ education > high school % (n)	43% (14)	27% (9)	30% (10)	33/72
Fathers’ education > high school % (n)	42%(11)	27% (7)	31% (8)	26/72

Mothers’ and fathers’ education levels (coded as 1 = primary school, 2 = middle school, 3 = high school, and 4 = university) were then correlated with the WPPSI global score. A positive and moderate correlation emerged in the Non-Verbal Profile between the mothers’ education levels and the WPPSI global score. The correlations are given in Table 9.

**Table 9:** Correlations between parents’ education levels and WPPSI global score by group.

	Verbal	Non-verbal	Homogeneous
Mothers’ education	0.130	0.307	0.060
Fathers’ education	0.042	0.283	0.137

*** < .001, ** < .01, * < .05.

### Developmental trajectories

Finally, descriptive statistics for each group are given in Table 10, with the results of the ANOVAs comparing the three groups on each milestone.

**Table 10:** Descriptive statistics for age (in months) when developmental milestones were reached, and results of the comparisons between the groups.

Group	Verbal	Non-verbal	Homogeneous	F	p	η_p_^2^
**Sitting**						
n	26	20	18	1.75	0.18	0.05
M (DS) [min–max]	8.83 (2.80) [5.5–14]	12.38 (11.74) [6–60]	8.94 (2.61) [5–13]			
**Babbling**						
n	24	20	16	4.22	0.02	0.13
M (DS) [min–max]	15.96 (6.12) [6–30]	21.25 (16.62) [5–78]	11.03 (3.55) [6–20]			
**Walking**						
n	27	22	21	1.48	0.24	0.04
M (DS) [min–max]	24.11 (5.54) [13–36]	27.41 (13.40) [12–72]	23.01 (5.01) [14–36]			
**First words**						
n	26	22	21	1.16	0.32	0.03
M (DS) [min–max]	27.54 (16.72) [12–96]	31.36 (13.85) [7–66]	24.71 (11.46) [10–60]			

*F* F-test value; *p*  significance level; *η*_*p*_^*2*^ eta partial squared expressing the effect size.

The descriptive statistics show that the Non-Verbal Profile group reached each milestone later than the other two groups, although the difference was only significant for babbling (F(2,57) = 4.22, p = 0.02, η_p_^2^ = 0.13), where the Non-Verbal Profile group reached this milestone later than the Homogeneous Profile group (t = 2.89, p = 0.02, d = 0.81). The age of reaching all milestones were then correlated with the total WPPSI score: sitting, babbling, walking, and first words correlated negatively and moderately with the global score in the Non-Verbal Profile group. All the correlations are given in Table 11.

**Table 11:** Correlations between age of reaching developmental milestones and WPPSI global score in each group.

	Verbal	Non-verbal	Homogeneous
Sitting	0.028	−0.652**	0.035
Babbling	−0.151	−0.675**	−0.233
Walking	−0.222	−0.572*	0.060
First words	−0.242	−0.385	−0.108

*** < .001, ** < .01, * < .05.

## Discussion

This study explored the cognitive profile of DS, focusing on verbal and non-verbal indices, as assessed with the WPPSI III, in an effort to provide further evidence on interindividual variability.

Looking at the cognitive profile of our sample as a whole data suggested a homogeneous picture, with no differences between participants’ verbal and non-verbal indices. The profile of our sample was not only homogeneous in terms of their verbal and non-verbal indices, but also when subtests were considered separately. It is noteworthy that participants’ expressive and receptive vocabulary (measured with the Picture Naming and Receptive Vocabulary tasks, respectively) did not differ, in contrast with previous reports e.g.,^51^. This discrepancy might be due to the different assessment methods used, however. In the present study, expressive and receptive vocabulary was assessed directly with the child, and measures of the developmental level were considered. In the study by Deckers et al.^6^, vocabulary was assessed from a parent's report, and by comparing the number of words understood and pronounced by the child. In the non-verbal domain, the scores obtained in the two subtests comprising the index did not differ, in line with the findings of Burgoyne et al.^52^.

That said, a marked interindividual variability emerged within our sample as a whole, revealing three subgroups of much the same size with different cognitive profiles. In fact, only one group of 21 participants had similar verbal and non-verbal skills (the Homogeneous Profile group), and obtained higher global cognitive scores than the other two subgroups. A second group of 22 participants (the Non-verbal Profile group) had lower scores in the verbal than in the non-verbal domain. This group of participants showed the lowest cognitive level. A third group of 29 children (the Verbal Profile group) obtained better results in the verbal domain (with scores as high as in the Homogeneous Profile group) than in the non-verbal one (their scores being similar to those of the Non-Verbal Profile group). These results explain the marked interindividual variability identified, both within groups and between different studies, suggesting that it would be better to assume that individuals with DS can express not just one, but multiple different cognitive profiles. One of our three subgroups (the Non-Verbal Profile group) had the features of the profile mostly described in the literature, with a better performance in non-verbal processing than in the verbal domain. Some previous studies had also found evidence of a profile characterized by better verbal than non-verbal scores e.g.,^20,22^, however, while others had reported finding similar scores in the two domains e.g.,^23^. As for the whole group, further analyses conducted at subtest level generally indicated homogeneity between subtests referring to the same index.

Our findings tend to be in line with the report from Tsao and Kindelberg^26^ on the only study that previously explored the possible existence of different cognitive profiles in DS. Focusing on childhood, they identified four profiles, three of which correspond to those emerging in our study. Their group with similar scores for verbal and non-verbal processing coincides with our Homogeneous Profile group, with one exception: whereas the scores obtained in the subtests of the two indices were much the same in our group, they saw a drastically worse score in one subtest (Classification, which was part of the Non-Verbal Index) than in the other. Their second group scored better in non-verbal subtests, like our Non-Verbal Profile group. Their third group obtained significantly higher scores in verbal subtests, like our Verbal Profile group. We found no cluster corresponding to their fourth group, which featured verbal scores close to the mean, and lower than non-verbal scores. This difference might be due to the tasks used to assess verbal and non-verbal skills, as some were similar (e.g., their “Vocabulary” task corresponded to our “Picture Naming” task), while others differed (e.g., none of the tasks we administered resembled their “Social comprehension” task). Another possible explanation would concern environmental variables that might have shaped participants’ cognitive profiles differently.

We examined three variables potentially influencing our sample’s different cognitive profiles. Medical conditions showed no association with the different profiles, although congenital heart defects are known to alter blood oxygenation, which affects brain development^53^, and OSA can impair consolidation processes during sleep^54^. Parents’ education levels were also unassociated with the different profiles, although this variable is known to modulate the cognitive level of individuals with DS^21,54,55^. It is worth noting, however, that parents’ education, when not considered as a dichotomous variable, showed stronger associations with the global scores obtained in the Non-Verbal Profile group than with those of our other two subgroups. This might give the impression that parents’ education level had more impact on the former group’s profile, while therapies could have had a confounding effect on this variable in the latter two groups. Regarding the third variable considered in this study, age on reaching developmental milestones, our Non-Verbal Profile group reached each milestone (and babbling in particular) at an older age than the other two groups, and it was the only group in which global cognitive scores correlated with age on reaching milestones. Since our Non-Verbal Profile group had the “classical profile” and the lowest global task performance scores, and reached developmental milestones later in life, we might surmise that this was the group in which genes had the most impact on the individuals’ cognitive profile. Alternatively, the higher scores obtained in the verbal domain by the Verbal Profile group or in both domains by the Homogeneous Profile group might be due to an enriched home environment and/or early therapeutic intervention. It has been widely suggested that the type of environment influences children’s cognitive development, with a “poor” environment leading to a “loss” of several IQ points, while a “rich”, stimulating environment and appropriate intervention can raise a child’s IQ score e.g.,^56^. These are mere speculations, of course, that would need to be tested in future studies.

None of the above-mentioned factors are independent of each other; they interact continuously, contributing to the variability seen in individuals with DS. From early infancy, nature and nurture interact and influence a child’s development in subtle ways e.g.,^57^. Early experiences are thought to initiate “developmental cascades”^58^ that, though difficult to monitor, shape the way infants respond to their environment. It is not that genes create an individual, and then this individual is influenced by the environment; there are multi-directional interactions constantly underway between the environment, the genetic material, and the individual^58^. Taking a multi-level approach and considering the variability at each level, we are therefore bound to find many factors influencing the DS phenotype. Future studies might aim to clarify which factors have a major role in shaping the cognitive variability involved in this syndrome.

While the present study contributes important information on the cognitive heterogeneity of children and adolescents with DS, it has some limitations that should be borne in mind. First, as is generally the case in neurogenetic syndrome research, the sample size is smaller than would be ideal for cluster analysis, and the sample has been recruited through a unique clinical center. It would be useful to replicate our findings in a larger sample, and to consider more variables (e.g., the quality of therapies, the characteristics of home environments) to enable an external validation of the clusters.

Finally, although we are aware that our study was only exploratory, our findings induce us to recommend taking the possibility of different cognitive profiles in DS into account in order to propose targeted interventions for children and their families. Such interventions need to be informed by an understanding of the emerging profile of a given individual with DS, enabling practitioners to focus on their strengths and thereby counter their weaknesses.

## Data Availability

The data that support the findings of this study are available upon request from the authors.
